# Allostatic Load and Exposure Histories of Disadvantage

**DOI:** 10.3390/ijerph18147222

**Published:** 2021-07-06

**Authors:** Lucy Prior

**Affiliations:** School of Geographical Sciences, University of Bristol, Bristol BS8 1SS, UK; lucy.prior@bristol.ac.uk

**Keywords:** neighbourhood deprivation, social capital, allostatic load, biosocial, health inequalities

## Abstract

The stress pathway posits that those in disadvantaged circumstances are exposed to a higher degree of stressful experiences over time resulting in an accumulated biological burden which subsequently relates to poorer health. Trajectories of disadvantage, in the form of neighbourhood deprivation and structural social capital, are evaluated in their relation to allostatic load representing the cumulative “wear and tear” of chronic stress. This paper uses data from the British Household Panel Survey and Understanding Society in a latent class growth analysis. We identify groups of exposure trajectories over time using these classes to predict allostatic load at the final wave. The results show that persistent exposure to higher deprivation is related to worse allostatic load. High structural social capital over time relates to lower allostatic load, in line with a stress buffering effect, though this relationship is not robust to controlling for individual sociodemographic characteristics. By demonstrating a gradient in allostatic load by histories of deprivation, this analysis supports a biological embedding of disadvantage through chronic exposure to stressful environments as an explanation for social health inequalities.

## 1. Introduction

The persistence of health inequalities across contexts and scales means understanding the processes of exposure-health relationships is an important area of research. Biosocial perspectives on health geography offer new avenues for investigating how gradients of disadvantage manifest in the health of bodies [1,2,3]. Concerned with the dynamic entanglements of social and biological processes, biosocial research can give insight into how environments “get under the skin” [4]. That is, biosocial data provides objective measures of the biological embedding of multiple exposures [5].

Pathways related to stress are relevant processes for understanding the transition from exposure to health. The social and physical environments which characterize different places can be varyingly perceived as threatening or stressful [6,7]. For example, the disorder that may typify deprived areas is commonly theorized to impact health through the incitation of stress [8,9,10]. Repeated exposure to such stressful environments results in “wear and tear” on the body and this weathering can negatively influence health, a process captured through the concept of allostatic load [11,12,13]. Moreover, other experiences may impart a stress-buffering influence, working to alleviate the negative impact of disadvantage. For example, the beneficial health effects of green space are often linked to stress reduction [14,15,16]. The stress-buffering hypothesis is also a major conceptual underpinning for positive associations of social capital with health [17,18]. These ideas feed into the so-called “stress pathway”, a biosocial mechanism to understand how different exposure histories are embodied over time in the health of individuals.

Investigating how exposures relate to later health states is a vital component to understanding health inequalities. The biosocial viewpoint, appreciating the importance of heterogeneous exposures and processes, allies with another major health concept: the exposome [19,20]. The exposome, designed as a conceptual complement to the genome, is focused on environmental exposures: considering the “environment” to encompass factors within and outside the body. Hence, the exposome is clearly aligned with biosocial ideas [3]. Similar to a biosocial lens on health, the dynamism of exposure and mutability of the body is central to the exposome. It considers the whole lifecourse and places exposures within a space-time framework of trajectories, rather than as static factors [21]. Therefore, it is a useful framework to investigate the stress pathway, where repeated exposure to situations perceived as stressful is vital to the allostatic process [5].

To investigate how stress-related exposures relate to a cumulative marker of biological weathering, it is therefore important to consider long-term environmental histories, appreciating the changing nature of exposure. This study will identify trajectories of neighbourhood deprivation and social capital over a 20-year period and relate these histories to allostatic load. This analysis offers a test of the chronic accumulation theory of the stress pathway through the lens of a biosocial and exposomic conceptual framework. Specifically, in line with expectations from the literature on the stress pathway hypothesis, this study aims to address the following research questions: does greater exposure to deprived neighbourhoods over time relate to higher allostatic load, and does experiencing higher social capital over time relate to lower allostatic load?

### Background

The stress pathway has long been posited as a critical element of individual outcomes in social health research. Previously this tended to be implicit, with a stress mechanism acting as an underlying theoretical proposal for explaining associations. For example, the income inequality hypothesis relies on conceptualising relative deprivation as a source of chronic stress to explain its relevance to health gradients [22,23]. Increasing availability of biodata within social surveys means a growing number of studies are explicitly investigating stress-related pathways. For example, studies have shown differences in cortisol levels and reactivity by the intensity of neighbourhood disadvantage, social control and poverty [24,25,26]. Dowd et al. [27] reviewed studies examining associations of socioeconomic status with cortisol and allostatic load. Overall, they found inconsistent evidence for associations with different cortisol measures. The labile nature of cortisol problematizes measurement [27]. In contrast, more agreement was found in relationships of socioeconomic status and allostatic load, which summarizes a long-term, accumulative response to stress [13,27].

Allostatic load is a prominent concept drawn upon in the burgeoning biosocial literature. Fitting with the “weathering hypothesis” [28], allostatic load captures the cost of chronic stress, with health implications for a variety of biological systems [12,13]. As a concept it reflects persistent exposure to stressful stimuli and the resultant physiological processes, but also the impact of behavioural habits and developmental processes that pattern exposure responses [12]. Allostatic load provides a useful tool in explaining social health inequalities over the lifecourse. For instance, Geronimus et al. [29,30] drew on the theorized framework of allostatic load in evidencing accelerated biological ageing through exposure to perceived stress, poverty, and negative social interactions.

Combining information on biomarkers from across physiological systems enables allostatic load to be operationalized in quantitative social research. In this way, allostatic load has been corroborated as predictive of mortality and a variety of morbidities [31,32,33,34]. A number of studies investigate how allostatic load relates to measures of socioeconomic status, proposing allostatic load as a biosocial link between social and health gradients. Johnson et al. [35] reviewed 26 studies, and found that, while the operationalisation of allostatic load varied in calculation method and biomarkers used, there was general consensus in low socioeconomic status relating to worse allostatic load. Therefore, higher allostatic load, representing a greater biological burden of chronic stress, would be expected for individuals with more ‘stressful’ disadvantaged exposure histories.

Recent work has explored biosocial pathways that may explain the “black-box” of how neighbourhoods influence health. The neighbourhood effects research paradigm has long called for exploration of the mechanisms of effects [36], with recent calls to pay particular attention to biosocial pathways and explanations for neighbourhood-health associations [3]. Studies have largely substantiated the conceptual framework of the stress pathway in relation to neighbourhood socioeconomic status, poverty, segregation, as well as social and physical environment “riskscapes” [37,38,39,40,41,42,43,44]. Recent work has further corroborated the biosocial processes of the stress pathway, providing evidence that allostatic load mediated relationships between neighbourhood deprivation and health [45]. However, as highlighted in a review by Ribeiro et al. [46] the majority of studies examining allostatic load and contextual exposures are cross-sectional in nature and many rely on the same datasets from the USA, limiting generalisability across different national contexts where particular societal conditions can produce different patterns of association [47].

Longitudinal data is a vital resource in understanding health pathways, helping to establish the temporal ordering of exposure then outcome and rule out alternative explanations such as selection effects [48]. Longitudinal studies that consider longer multi-year to decadal time frames are also important in enabling a wider variety of research questions concerning lifecourse hypotheses and exposure-health trajectories. For example, following a history of developmental research, such as that on the foetal origins hypothesis [49,50], a variety of early-life experiences have been shown to have long-standing influences on later-life biomarkers. Barboza Solís et al. [51,52] found associations of adverse childhood experiences and socioeconomic position with allostatic load at 44-years-old in the 1958 British birth cohort. Using retrospective reports, Friedman et al. [53] evidenced an association between early-life adversity and allostatic load later in life. Similarly, Non et al. [54] found social adversity assessed in childhood was significantly associated with cardiometabolic risk in mid-life. These studies suggest an early-life biological embedding of disadvantage with long-term consequences for health inequalities.

Moreover, research has also explored the contribution of different lifecourse hypotheses for the relationship of social status and health over time. For example, Walsemann et al. [55] investigated a set of lifecourse models, such as sensitive period and accumulation, for the association of socioeconomic status in adolescence and adulthood with biomarkers of cardiovascular risk. They found support for each of the lifecourse hypotheses varied by gender and ethnicity: for example, all models were supported for white women, whereas they were unable to demonstrate the influence of any of the models among black participants. Additionally, Yang et al. [56] showed direct and indirect pathways from early-life socioeconomic status to biomarker summaries of inflammatory and metabolic burdens, as well as finding evidence for an accumulative impact of disadvantage. A potential sensitive period at the transition to adulthood was demonstrated by Gustafsson et al. [57] for the influence of social adversity on mid-life allostatic load, with an accumulative model also supported.

An accumulative impact of disadvantage over time is a common model for linking social and health inequalities, and one which fits well with allostatic weathering as a representation of the total cost of adapting to the environment over time [5]. Lifecourse accumulation models exploring neighbourhood conditions are rare, given the operational difficulties of collecting or linking geographic data over long histories. Lemelin et al. [58] obtained 20-year residential histories for participants, creating measures of average exposure to neighbourhood poverty over time. They found greater cumulative exposure was associated with a biomarker of subclinical atherosclerosis, but only for women [58]. Another example used Swedish cohort data to demonstrate how cumulative neighbourhood disadvantage throughout the lifecourse significantly predicted higher allostatic load in mid-life [59]. However, the sporadic and sometimes unclear direction and strength of relationships means further research exploring associations of neighbourhood-level circumstances and biomarkers over long time periods is still needed. In particular, following the framework provided by the exposome, exploring exposure trajectories would facilitate insight into the biological embedding of stressors.

In addition to the consideration of contextual exposures, there are also more limited studies which analyse aspects of social capital in relation to biosocial mechanisms. In view of the entanglement of social capital with stress-related theorisations this is a gap which needs addressing. Robinette et al. [9] drew upon the neighbourhood health literature in showing that perceptions of neighbourhood cohesion predicted a biomarker summary of cardiometabolic risk four years later. Psychosocial processes of social support and isolation were also implicated in work by Stafford et al. [60], who showed that older persons who had recently become widowed or newly living alone had higher night-time cortisol levels that those married or living with others, respectively. However, both these studies have relatively short time frames and only two points of social capital or support data. In a study of childhood maltreatment, Horan and Widom [61] found that lower perceived social support throughout the life span was related to higher allostatic load and partially mediated the association of maltreatment with allostatic load. Capitalising on the social data resource of longitudinal studies to explore the dynamics of social capital over long-time periods and their relationship to biodata can clearly contribute to understandings of stress-related health pathways. This study contributes to this literature in evaluating whether there is evidence to support a stress-buffering model of social capital and health through a biosocial lens.

The dynamics of environmental exposure are of central concern in exposome studies [19]. This points towards thinking about exposure to different trends of factors over time. Analysing exposure trajectories can further our understanding of health inequalities through appreciating heterogeneity in heath states between those who have experienced a dynamically changing environment and those with a more static exposure history. Variety in trajectories can also be exploited to explore lifecourse models. For example, Gruenewald et al. [62] compared the degree of allostatic load between trajectories of socioeconomic status from childhood to adulthood. They reported that those with persistently low status had the highest allostatic load, suggesting a cumulative association, followed by those experiencing a downward trend in status, potentially indicating a negative impact from loss of status [62]. Lin et al. [63] reported that older persons with consistently high socioeconomic position over their lifecourse had significantly lower levels of two inflammatory biomarkers than those who had constantly low status, or those who had experienced upward social mobility. Therefore, a model of social mobility may not always impart a biological health benefit [63]. However, both these studies rely in part on retrospective reporting which can introduce bias. Studies which investigate trajectories of multiple exposures measured across a series of timepoints would be a valuable contribution to the literature on health inequalities. Moreover, the social sphere is largely underrepresented in exposome research currently, meaning studies of dynamic exposure histories and their relation to biosocial processes are needed.

This analysis investigates the stress pathway by examining how long-term exposure histories of neighbourhood deprivation and social capital relate to later allostatic load. A latent class approach will be used, accounting for heterogeneous trajectories in unobserved (latent) sub-groups of the population. It is hypothesized that the identified exposure trajectories will follow graded associations with allostatic load. Higher or worsening deprivation is expected to be related to increased allostatic load, in comparison to trajectories that reflect less disadvantaged histories. According to the stress-buffering hypothesis, higher or increasing social capital, in comparison with lower or decreasing social capital, is anticipated to be associated with lower allostatic load.

## 2. Materials and Methods

Data for this analysis is drawn from the British Household Panel Survey (BHPS) and the follow-on UK Household Longitudinal Study (UKHLS commonly referred to as Understanding Society) which as well as enrolling new participants continued to sample consenting BHPS participants from Wave 2 onwards [64]. At Wave 3 of Understanding Society (collected between 2011 and 2012) a nurse-based health assessment was carried out for eligible participants of the BHPS sample, taking a blood sample from which a range of biomarkers could be extracted [65,66,67]. Our sample consists of 3210 individuals who had non-missing information on at least one of the biomarkers used to construct allostatic load.

The response is allostatic load, marking physiological weathering due to chronic stress exposure. An index is constructed from 13 biomarkers (see Table 1), encompassing measures from multiple physiological systems. The index is a summary risk-score, counting the number of biomarkers for which participants fell into high-risk quartiles (this was the lowest quartile for DHEAs, HDL cholesterol and albumin, elsewise the highest quartile). Quartile cut-offs are presented in Table 1; though sample-based these cut-offs correspond well to clinical cut-points, where these are known for the biomarkers [65]. This operationalisation follows previously established conventions in constructing allostatic load measures [12,34].

Townsend deprivation scores [68] are used to construct neighbourhood disadvantage exposure histories. The Townsend index is calculated based on four measures: unemployment; non-car ownership; non-home ownership; and household overcrowding. Z-scores are calculated for the percentage of each of the four measures within small-area units (logged percentages are used for unemployment and overcrowding to account for skew). The Townsend deprivation score is the sum of these z-scores. Positive Townsend deprivation scores indicate more deprived areas, whilst negative values represent relatively less deprived areas than average.

Townsend deprivation scores are derived from the 1991, 2001 and 2011 UK Censuses, and harmonized to 2011 Lower Layer Super Output Areas (LSOAs), providing a time-comparable index (for details on the harmonisation methodology see [69,70,71]). The 1991, 2001 and 2011 Townsend scores and quintiles linked with the 2011 LSOA codes were provided to the author by Paul Norman. Scores are matched to the main dataset by 2011 LSOA or DZ code [72,73]. For the BHPS waves we first had to match the 2001 LSOA and DZ codes to their 2011 counterparts. A simple approach is taken, keeping those LSOAs in England and Wales that were unchanged between 2001 and 2011 (97% of areas in the sample) [74], and for Scotland we kept those areas where the 2001 centroid fell inside the 2011 boundary (95% of Scottish DZs in our sample) [75,76].

To account for change in deprivation over time, the Townsend deprivation scores are linked to every other wave of the BHPS, and additionally to Wave 2 of Understanding Society, creating 10 timepoints of exposure history. The scores were applied to the 10 timepoints treating census years as mid-points: thus, 1991 Townsend deprivation scores were assigned to BHPS Waves 1, 3 and 5; 2001 scores to BHPS Waves 7, 11 and 13; and 2011 scores covered the final 4 timepoints (BHPS Waves 15 and 17 and Wave 2 of Understanding Society).

Participants were asked whether they joined in the activities of any of a list of organisations on a regular basis, whether or not they were formally a member of those organisations. The list of potential organisations included 16 organisations, such as “Political party”, “Trade unions”, and “Environmental group”. The full list can be accessed online (www.understandingsociety.ac.uk, accessed on 9 May 2021). We use information on this variable from every other wave of the BHPS (from wave 1 to 17 inclusive) and additionally from wave 3 of Understanding Society. For each of these 10 timepoints, a summary count measure of the number of organisations respondents identified as regularly active in was calculated, providing a history of structural social capital. The social capital variable ranged between 0 and 9.

To account for sociodemographic characteristics important to relationships of chronic stress and health, a series of covariates measured contemporaneously with the biomarker data are assessed when predicting allostatic load. Age and sex are included, as well as education, employment status, tenure, marital status and subjective financial situation. Age is a continuous variable, centred around the mean of 51.5 years-old, whilst all other variables are categorical. Summaries of the covariates and allostatic load are presented in Table 2.

This analysis seeks to identify distinct trajectories of social capital and deprivation, and to evaluate how these histories relate to later allostatic load. For the first stage of this process—identifying trajectories of exposure—this analysis uses latent class growth analysis (LCGA). LCGA is a method for modelling the change in a variable allowing for different trajectories across sub-groups of the population [77]. These sub-groups are unobserved, capturing interindividual heterogeneity through latent classes.

To identify distinct exposure histories a set of LCGA models are run for social capital and Townsend deprivation, specifying an increasing number of latent groups, building upwards from 2 classes. Each model run is compared using model fit and other indices to determine the most appropriate number of classes. For Townsend deprivation, the latent classes are defined based on data for 3095 individuals, for social capital the trajectories are based on 3096 individuals. Panel membership across the timepoints can vary, resulting in an unbalanced panel which is estimated using full information maximum likelihood. For sensitivity analysis of selection bias, the analysis was repeated with a fully balanced panel of 1177 individuals. Results are presented in Supplementary Materials.

The second stage of the analysis involves investigating how the exposure histories relate to allostatic load, the distal outcome. This analysis uses a three-step approach which involves: (1) estimation of the latent classes; (2) assignment of individuals to the different classes based on posterior class membership probabilities; and (3) use of latent class memberships as observed variables in predicting the response of interest [78,79,80]. An adjusted version of the three-step method proposed by Bolck, Croon and Hagenaars [81], which we will refer to as the BCH method, is employed to account for potential bias and attenuation of estimates that can be introduced through the classification procedure [79,81]. This method avoids shifts in the definition of classes; at the final step the classes are known [78,82]. The BCH method has been shown to perform well in comparison to one-step (where identification of the classes and their association to distal outcomes is simultaneously estimated), standard three-step and other corrected three-step approaches [78,79].

A series of models using the BCH method are implemented to assess relationships of deprivation and social capital exposure classes to allostatic load. Firstly, we run a null model where only the latent classes are used to predict allostatic load. Secondly, a model is run controlling for the key demographic characteristics of age and sex. Finally, a full model containing all socioeconomic covariates is tested to see whether the exposure trajectories influence allostatic load beyond the impact of more proximal stress-related exposures.

Data preparation was carried out in Stata version 15 [83]. The LCGA and BCH method analysis was conducted using Mplus version 7 [82,84].

## 3. Results

The first stage of the main analysis involved identifying an appropriate number of latent classes to summarize the trajectories of neighbourhood deprivation and social capital. Table 3 presents the model comparisons for both exposure measures. Though the sample-size adjusted Bayesian Information Criteria (SSABIC) is smallest for the 6-class model, indicated a better fitting model, for social capital a three-class solution is deemed most appropriate as maintaining a larger sample size (>50) for each exposure trajectory is desirable. For deprivation, the four-class solution is chosen: the additional fifth class did not add a substantially different trajectory history, and the Lo, Mendell, and Rubin likelihood ratio test [85] returned a highly non-significant value showing the 5-class solution was not an improvement over the 4-class model. Entropy was reasonably close to 1 for both of the selected models indicated that the classes were well separated from each other. The classes for social capital and deprivation are presented in the Supplementary Materials. Note that for the Townsend score exposure histories a quadratic growth term is also included as this addition was found to improve model fit over a linear change formulation.

**Figure 1:**
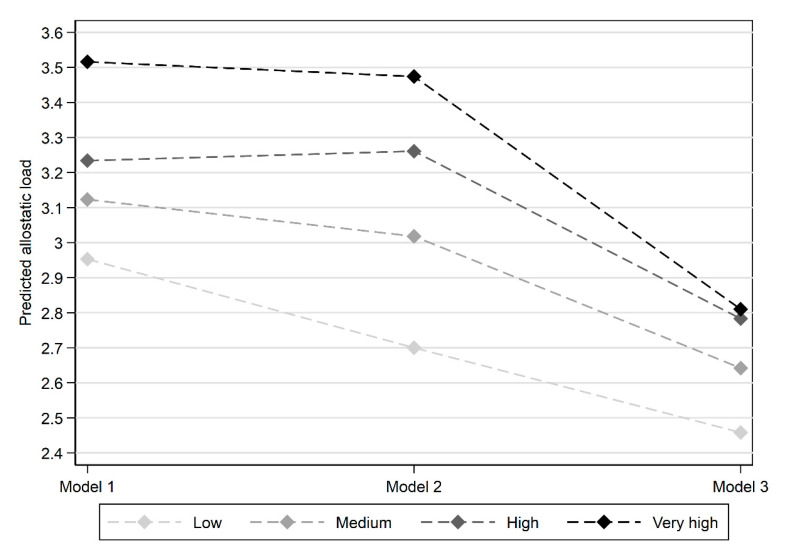
Mean predicted allostatic load score by deprivation history for each model.

The second stage of the analysis examined the relationship of allostatic load to the exposure histories of disadvantage and social capital. Figure 1 presents the mean allostatic load scores for each of the deprivation classes across the series of models. Evidence to support the first research question is provided in Figure 1: allostatic load is patterned by neighbourhood deprivation, with histories reflecting greater and more severe exposure to disadvantage associated with higher allostatic load. The overall difference between classes decreases as sociodemographic characteristics are accounted for in Model 3. Indeed, the exposure histories are significantly related to allostatic load in Models 1 and 2, but the relationship borders on insignificance when more proximal characteristics are controlled for (see Table 4).

**Figure 2:**
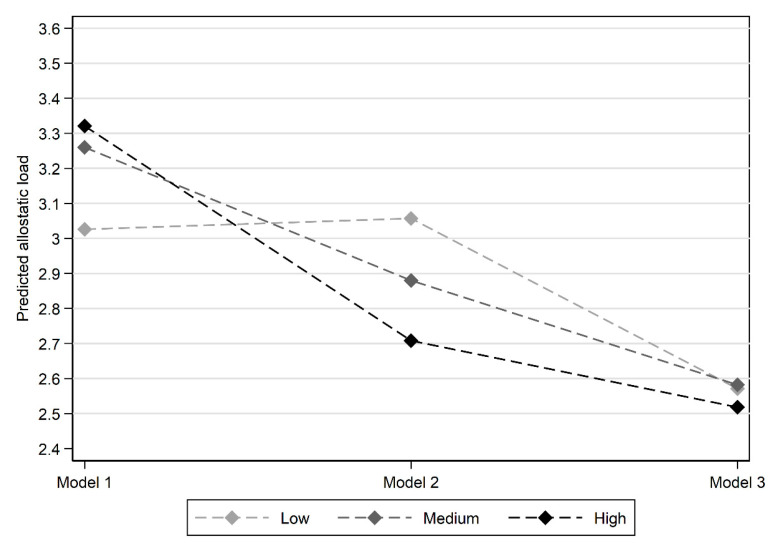
Mean predicted allostatic load score by social capital history for each model.

The results by trajectories of social capital are presented in Figure 2. Accounting for the influence of age and sex, and the other socioeconomic characteristics—Models 2 and 3—revealed those in the high trajectory of social capital exhibited the lowest allostatic load. In Model 2 there is a clear gradient across the social capital histories which is in agreement with a stress-buffering hypothesis—that is belonging to more organisations lowers allostatic load. However, the differences between the social capital classes are not significant at the 95% confidence level in Model 2 and become marginal and highly non-significant when the full range of sociodemographic characteristics are controlled for in Model 3 (see Table 5). Therefore, whilst some support is provided for the second research question in terms of the patterning of results—where lower allostatic load is associated with higher social capital trajectories—this support is limited regarding the significance and strength of the identified effects.

## 4. Discussion

This analysis aimed to explore the stress pathway as an explanation for social health inequalities. It did so through deriving and exploring the trajectories of neighbourhood deprivation and structural social capital that individuals from Great Britain experienced over a 20-year period. These trajectories were then related to later allostatic load to assess whether their associations with this marker of accumulated wear and tear on the body were in line with the theorisation of the stress pathway. The results indicated that heightened exposure to deprived environments was significantly associated with a higher chronic stress burden. However, more limited evidence was provided in support of a stress-buffering role for social capital, as the initial apparent association with allostatic load was reduced and become insignificant on control for the sociodemographic characteristics of individuals.

Drawing upon rich individual histories, we identified a four-class solution for Townsend deprivation exposure, reflecting reasonably consistent trajectories, summarising exposure at various degrees of severity. Each class also exhibited a small improvement over time, with a slight worsening of scores in the latter years. This could be a reflection of general trends in deprivation nationally. Norman [86] evaluated changes in Townsend scores in England harmonized between 1971 and 2011, and showed a general improving trend in deprivation, with a small increase to 2011 which they attribute to rising non-home ownership and unemployment.

The identified deprivation histories are indicative of relative stability in exposure over time. This stability represents both people remaining in place and individuals who move between neighbourhoods with similar environments. It is beyond the scope of the current analysis to explore these specific movements of people or to say exactly why the exposure histories appear so stable. However, previous literature shows that people are likely to remain in similar places over time [87,88]. The social structures of places are often slow to change, with persistent patterning of relatively advantaged and disadvantaged areas over long periods [89,90]. In addition, where individuals undergo a residential move, this does not usually involve a large differential in the type of place occupied [91]. We are cautious, however, of overstating any implications of the results for questions of social mobility and being “stuck in place” [92], or the “stickiness” of places and people [93]. The modelling strategy assumed homogeneity within classes (in other words, internal variance was restricted to zero) which may have limited our ability to delineate more dynamic trajectories which may be important but are less common. This simplified modelling strategy was beneficial to the identification of distinct exposure histories as it was computationally less intensive and more readily achieved model convergence. Application of a similar modelling strategy to identify exposure trajectories in contexts of more dynamic social change or where persons are more mobile, both geographically and socially, may be a profitable pathway for future research into understanding the mechanisms of health inequalities over time.

The main results offer support for the stress pathway theorisation. Trajectories which represented exposure to higher deprivation over time were associated with worse allostatic load, in comparison with classes reflecting more advantaged histories. This patterning was maintained throughout models which controlled for the influence of proximal stressors on allostatic load, though the strength of the association was lessened. The findings align with cross-sectional studies which have presented graded relationships between multidimensional measures of deprivation and allostatic load [46]. They also substantiate previous studies which have evidenced cumulative associations of disadvantage with allostatic load [58,59,62,63]. Health inequalities by social status continue to be persistent features of society [94,95,96]. By demonstrating a gradient in allostatic load by histories of deprivation, this analysis supports a biological embedding of disadvantage over time through chronic exposure to stressful environments as an explanation for these health inequalities. Therefore, in working to address health inequalities research would benefit from paying attention to the structural systems and cultural processes that work to persistently expose people to these ‘stressful’ environments over time. Work to investigate the particular environmental features which contribute to an enhanced biological burden over time could also help in revealing more proximal factors suitable for shorter-term interventions.

In addition to deprivation trajectories, this analysis also provided insight into the dynamics of a measure of structural social capital. Three classes were identified, capturing groups of individuals that: were active in very few organisations and whose social capital marginally declined over time; maintained a steady level of activity in between 1 and 2 organisations; and that had high levels of social capital and who increased their participation over time. Initially, a relationship contrary to expected was found, with a beneficial impact of advantaged social capital trajectories only revealed once age and sex were controlled for. Those with high structural social capital through activity in organisations are likely to be older, retired persons who have more time to contribute to multiple institutions [97]. Older persons are also more likely to present worse allostatic load due to the general decline in health functioning by age, hence the artificially elevated allostatic load of the high social capital group in Model 1.

This analysis contributed to the literature on the role of social capital in health patterns through exploring a long exposure history of organisational membership and its association with a biomarker summary of chronic stress. The patterning of the social capital histories with allostatic load in Models 2 and 3 broadly supported the stress-buffering hypothesis. It follows research showing that increased social participation over time, measured by whether participants became active in any organisations, was associated with improved self-rated health [98]. However, overall support cannot be provided for the association of structural social capital and allostatic load; the differences between latent classes were not significant. Other studies have also shown that structural measures of social capital may be less influential on health than cognitive measures. For example, Yip et al. [99] demonstrated relationships between cognitive social capital and several health measures, but did not evidence similar associations for social capital as captured through organisational membership. Similarly, Fujiwara and Kawachi [100] did not demonstrate an association of structural dimensions of social capital with depression at follow-up, whereas they did find relationships for social trust and belonging. It could be that cognitive dimensions of social capital, including aspects such as trust, support, and norms of reciprocity [101] are more relevant to counteracting stressful circumstances than more formal interactions with organisations. For instance, Riumallo-Herl et al. [102] found relationships of social support and trust with hypertension biomarkers consistent with theorisations of social capital as a stress moderator. Research which explores multiple dimensions of social capital and biomarkers is needed to further address their varying contribution to health pathways.

A strength of this study is the use of a dataset covering a long period over which to gather information on exposure to deprivation and social capital. However, it is important to recognise that as the sample is drawn from Great Britain our results may not be widely generalisable to other national contexts with differing cultural and social environments. For example, future research exploring the impact of exposure to disadvantage under situations of rapid social, health and economic transition would help to uncover further insight into the temporal dynamics of the stress pathway. Research could consider the impact on health of the dose and duration of different exposures, for instance. Previous studies using Chinese data have indicated that circumstances typically associated with higher socioeconomic status, rather than lower, can be associated with worse allostatic load [47], which indicates the continued need for studies across varying study contexts. Moreover, whilst this study benefitted from the presence of biodata for individuals, it was restricted to analysing a single timepoint. Multiple occasions of biomarker measurements would be helpful for future research as this would allow researchers to evaluate more dynamic associations between exposures and the mechanisms through which they ‘get under the skin’.

The deprivation exposure measure was focused on the neighbourhood unit, defined as LSOAs. This was deemed a sensible geographic scale at which to capture every day, residential exposure to deprived environments, and practical in allowing linkage to Townsend scores. However, these types of static, neatly bounded measures of neighbourhood have been criticised for their deficiencies in capturing the exposure of highly mobile persons [103,104,105]. Additionally, the ‘neighbourhood’ may not be the phenomenon scale [104] at which deprivation is most potent for allostatic load. Other environments and scales, such as the household, could be interesting avenues for future research to explore in studying the various dimensions of stress pathway as an explanation for social health inequalities. Furthermore, the measure of deprivation largely comprised structural aspects of disadvantage (unemployment; non-car ownership; non-home ownership; and household overcrowding): these may not be the most relevant aspects of deprivation in influencing the stressfulness of living in particular areas. Factors such as crime, access to green space or other resources, and social disorder may be more powerful environmental factors, for instance.

## 5. Conclusions

This study drew upon the framework of the exposome to examine dynamic exposure histories of disadvantage over time. By assessing two important social dimensions of disadvantage, deprivation and social capital, this analysis contributes a valuable insight into the social sphere of the exposome and how it relates to allostatic load. This analysis supported a model of the biological embodiment of disadvantage over time through chronic stress exposure, with persistent experience of highly deprived environments associated with worse allostatic load than exposure to more advantaged histories. In doing so we contribute support for a biosocial explanation of health inequalities.

This study demonstrates the value of evaluating environmental exposure histories over long time periods and highlights that exploring biosocial pathways for linking exposures to health may be fruitful avenues for developing understanding of the development of health inequalities. Future research would benefit from examination of exposure histories and their relation to biomarkers. In particular, there is clear scope to investigate more complex intra- and inter-individual heterogeneity in trajectories and to explore dynamic interactions between social exposures over time. This would help to reveal a more nuanced picture of exposure and biosocial health pathways.

## Figures and Tables

**Table 1:** Biomarker summaries.

System	Biomarker	N	Mean (SD)	High Risk Cut-Off Values
Cardiovascular	Systolic Blood Pressure	2628	126.44 (16.64)	≥136.5 mmhg
	Diastolic Blood Pressure	2628	73.01 (10.84)	≥80 mmhg
	Pulse Rate	2628	68.79 (10.93)	≥75.5 bpm
Lipid Metabolism	HDL Cholesterol	3138	1.53 (0.45)	<1.2 mmol/L
	Total: HDL Cholesterol	3137	3.75 (1.35)	≥4.42
	Triglycerides	3144	1.79 (1.27)	≥2.2 mmol/L
	BMI	3112	28.02 (5.52)	≥30.9 kg/m^2^
	Waist Circumference	3161	93.70 (14.52)	≥103.1 cm
Glucose Metabolism	HbA1c	2969	37.30 (8.67)	≥39 mmol/molhb
Inflammatory	C-Reactive Protein	3019	3.24 (6.60)	≥3.2 mg/L
	Fibrinogen	3121	2.81 (0.62)	≥3.2 g/L
	Albumin	3139	46.62 (2.94)	<45 g/L
HPA-axis	DHEAs	3137	4.74 (3.36)	<2.2 mol/L

**Table 2:** Summaries of allostatic load and sociodemographic characteristics from the final wave.

Factor		Mean (SD)	N
Allostatic load		3.07 (2.45)	3210
Age		51.53 (17.58)	3210
		**%**	
Sex	Female *	54.83	3210
	Male	45.17	
Education level	Higher *	31.29	3186
	Middle	46.39	
	Lower	22.32	
Employment status	Employed *	56.07	3210
	Retired	29.16	
	Unemployed/Inactive	14.77	
Tenure	Owned *	79.25	3206
	Privately rented	8.86	
	Socially rented	11.79	
Marital status	Married *	69.31	3210
	Single/SDW	30.69	
Subjective financial situation	Comfortable/Alright *	66.06	3209
	Just getting by	25.62	
	Finding it difficult	8.32	

Notes: * indicates reference category. ‘Higher’ education level includes degrees and other higher qualifications. ‘Middle’ education level includes A-Levels, GCSEs, or equivalents qualifications. ‘Lower’ education level includes other qualifications or none. ‘SDW’ stands for separated, divorced or widowed.

**Table 3:** LCGA model comparison with different numbers of classes (selected model highlighted in bold).

	Classes	SSABIC	Smallest Class Size		Entropy	LMR-LRT
			**%**	**Count**		
**Townsend** **deprivation**	**2**	**93,780.35**	**0.33**	1019	0.907	0.000
	3	88,670.63	0.14	425	0.892	0.000
	**4**	**86,475.98**	**0.08**	**246**	**0.879**	**0.002**
	5	85,530.94	0.05	147	0.844	0.276
	6	84,555.45	0.05	143	0.854	0.129
*Social capital*	2	58,948.61	0.18	543	0.898	0.000
	**3**	**57,478.44**	**0.07**	**203**	**0.826**	**0.092**
	4	56,809.16	0.02	48	0.808	0.021
	5	56,435.34	0.01	45	0.773	0.099
	6	56,189.13	0.01	46	0.761	0.362

**Table 4:** Estimated allostatic load means by deprivation history and covariate coefficients predicting allostatic load.

		Model 1: No Covariates		Model 2: Age and Sex		Model 3: Sociodemographics	
**N**		**3095**		3095		3067	
	Allostatic load	Mean	S.E.	Mean	S.E.	Mean	S.E.
Deprivation Exposure History	Low	2.953	0.072	2.700	0.081	2.458	0.108
	Medium	3.123	0.087	3.018	0.092	2.642	0.122
	High	3.234	0.109	3.261	0.108	2.783	0.140
	Very high	3.516	0.177	3.474	0.170	2.810	0.206
	Overall test *p*-value	0.015		0.000		0.050	
		Beta	S.E.	Beta	S.E.	Beta	S.E.
Age				0.053	0.002	0.052	0.004
Sex	Female *						
	Male			0.292	0.079	0.302	0.080
Education Level	Higher *						
	Middle					0.238	0.096
	Lower					0.463	0.123
Employment Status	Employed *						
	Retired					−0.054	0.140
	Unemployed/Inactive					−0.005	0.126
Subjective Financial Situation	Comfortable/Alright *						
	Just getting by					0.268	0.098
	Finding it difficult					0.478	0.170
Tenure	Owned *						
	Privately rented					0.265	0.150
	Socially rented					0.699	0.160
Marital Status	Married *						
	Single/SDW					−0.163	0.090

Notes: * indicates reference category. Robust standard errors accounting for clustering within the neighbourhood (LSOA) units are used.

**Table 5:** Estimated allostatic load means by social capital history and covariate coefficients predicting allostatic load.

		Model 1: No Covariates		Model 2: Age and Sex		Model 3: Sociodemographics	
**N**		**3096**		3096		3068	
	Allostatic load	Mean	S.E.	Mean	S.E.	Mean	S.E.
Social Capital Class	Low	3.026	0.060	3.057	0.066	2.571	0.114
	Medium	3.260	0.105	2.880	0.108	2.582	0.121
	High	3.321	0.177	2.708	0.180	2.518	0.189
	Overall test *p*-value	0.072		0.087		0.950	
		Beta	S.E.	Beta	S.E.	Beta	S.E.
Age				0.053	0.002	0.051	0.004
Sex	Female *						
	Male			0.277	0.079	0.300	0.080
Education Level	Higher *						
	Middle					0.244	0.100
	Lower					0.501	0.129
Employment Status	Employed *						
	Retired					−0.051	0.140
	Unemployed/Inactive					−0.003	0.126
Subjective Financial Situation	Comfortable/Alright *						
	Just getting by					0.293	0.098
	Finding it difficult					0.518	0.170
Tenure	Owned *						
	Privately rented					0.280	0.149
	Socially rented					0.803	0.156
Marital Status	Married *						
	Single/SDW					−0.143	0.090

Notes: * indicates reference category. Robust standard errors accounting for clustering within the neighbourhood (LSOA) units are used.

## Data Availability

Data are available through the UK Data Archive at: DOI:10.5255/UKDA-SN-7251-3, SN: 7251; DOI:10.5255/UKDA-SN-6136-2, SN: 6136; DOI:10.5255/UKDA-SN-6614-1, SN: 6614; DOI:10.5255/UKDA-SN-7248-7, SN: 7248.

## Supplementary Materials

The following are available online at https://www.mdpi.com/article/10.3390/ijerph18147222/s1, Figure S1: Exposure histories for Townsend deprivation score and structural social capital, Table S1: Estimates allostatic load means by deprivation histories and covariate coefficients predicting allostatic load for balanced sample of BHPS participants, Table S2. Estimated allostatic load means by social capital histories and covariate coefficients predicting allostatic load for balanced sample of BHPS participants.
