# Supplementary material for: Allostatic Load and Exposure Histories of Disadvantage
Source: Int J Environ Res Public Health. 2021 Jul 6;18(14):7222. doi: 10.3390/ijerph18147222 (PMC8308019; doi:10.3390/ijerph18147222)
Supplement: Supplementary file 1 [file ijerph-18-07222-s001.zip › ijerph-1234875-supplementary.pdf]

International Journal of
Environmental Research and Public Health

MDPI

Allostatic Load and Exposure Histories of Disadvantage-Supplementary Information

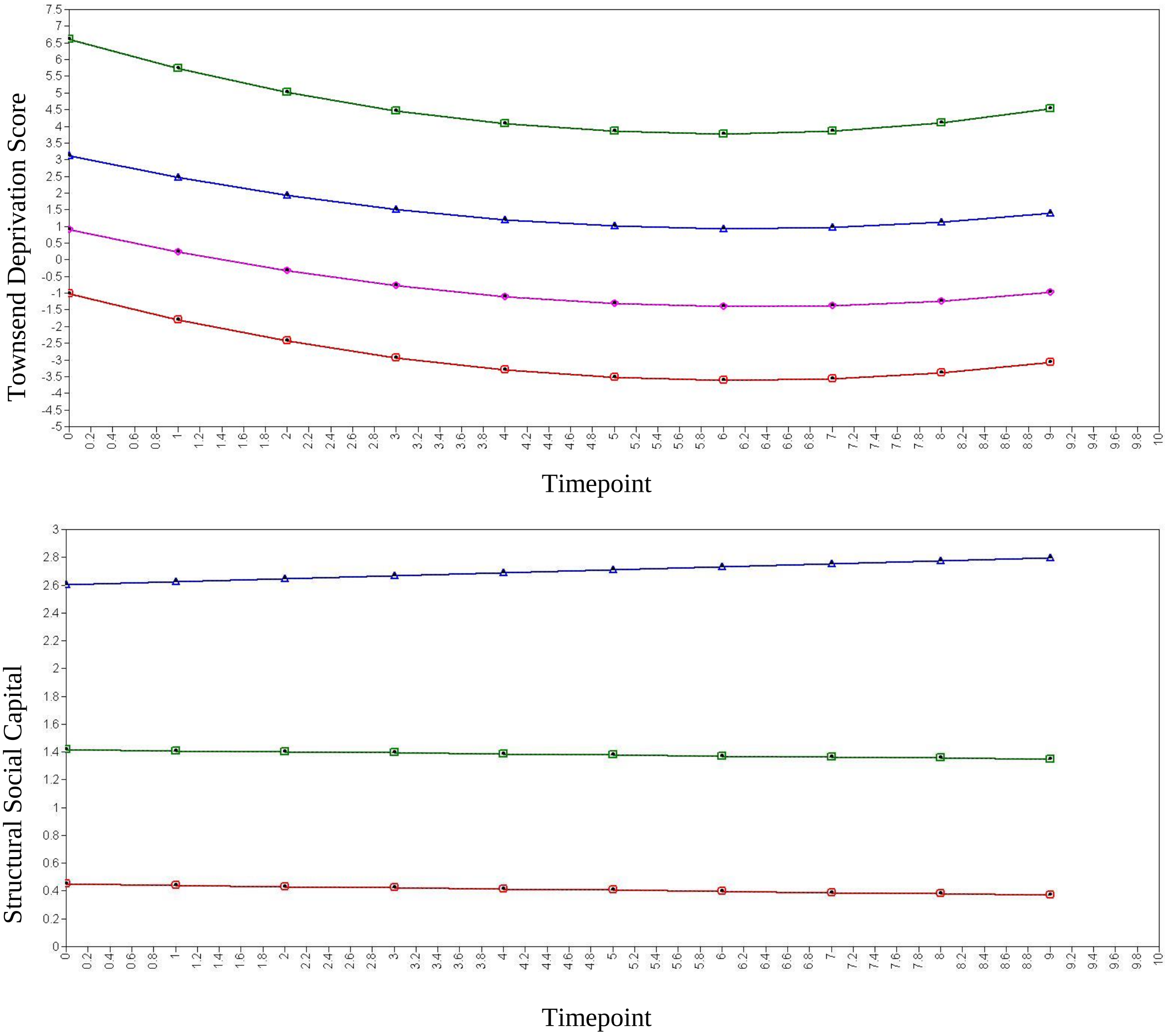

Figure S1. Exposure histories for Townsend deprivation score and structural social capital.

Table S1. Estimated allostatic load means by deprivation gistories and covariate coefficients predicting allostatic load for balanced sample of BHPS participants.

		Model 1: No covariates		Model 2: Age and sex		Model 3: Sociodemographics	
N		**1177**		1177		1175	
	Allostatic load	Mean	S.E.	Mean	S.E.	Mean	S.E.
Deprivation Exposure History	Low	3.300	0.102	3.202	0.112	2.741	0.183
	Medium	3.525	0.144	3.482	0.150	2.913	0.228
	High	3.818	0.181	3.793	0.187	3.129	0.261
	Very high	3.678	0.306	3.567	0.305	2.816	0.368
	Overall test p-value	0.067		0.022		0.293	
		Beta	S.E.	Beta	S.E.	Beta	S.E.
Age				0.043	0.005	0.034	0.008
Sex	Female*						
	Male			0.162	0.137	0.226	0.139
Education	Degree*						
	A-Level/GCSE					0.267	0.094
	Other/None					0.254	0.166
Employment Status	Employed*						
	Retired					0.267	0.094
	Unemployed/Inactive					0.219	0.222
Subjective Financial Situation	Comfortable/Alright*						
	Just getting by					0.373	0.303
	Finding it difficult					0.255	0.175
Tenure	Owned*						
	Privately rented					0.554	0.297
	Socially rented					0.191	0.384
Marital status	Married*						
	Single/SDW					0.131	0.156

Notes: * indicates reference category.

Table S2. Estimated allostatic load means by social capital histories and covariate coefficients predicting allostatic load for balanced sample of BHPS participants.

		Model 1: No covariates		Model 2: Age and sex		Model 3: Sociodemographics	
N		1177		1177		1175	
	Allostatic load	Mean	S.E.	Mean	S.E.	Mean	S.E.
Social Capital Class	Low	3.405	0.099	3.399	0.112	2.641	0.222
	Medium	3.581	0.137	3.464	0.148	2.892	0.194
	High	3.516	0.246	3.230	0.251	2.758	0.282
	Overall test p-value	0.607		0.712		0.397	
		Beta	S.E.	Beta	S.E.	Beta	S.E.
Age				0.042	0.005	0.030	0.008
Sex	Female*						
	Male			0.157	0.138	0.232	0.139
Education	Degree*						
	A-Level/GCSE					0.328	0.099
	Other/None					0.322	0.174
Employment Status	Employed*						
	Retired					0.328	0.099
	Unemployed/Inactive					0.276	0.222
Subjective Financial Situation	Comfortable/Alright*						
	Just getting by					0.389	0.299
	Finding it difficult					0.292	0.171
Tenure	Owned*						
	Privately rented					0.630	0.297
	Socially rented					0.174	0.381
Marital status	Married*						
	Single/SDW					0.194	0.157

Notes: * indicates reference category.
